# P049. Migraine influence on female reproductive life and motherhood

**DOI:** 10.1186/1129-2377-16-S1-A74

**Published:** 2015-09-28

**Authors:** Matteo Fuccaro, Cristina Martini, Martina Bruno, Matteo Bellamio, Federico Mainardi, Carlo Lisotto, Giorgio Zanchin, Ferdinando Maggioni

**Affiliations:** Department of Neurosciences, Headache Centre, University of Padua, Padua, Italy; Headache Centre, Hospital SS. Giovanni and Paolo, Venice, Italy; Headache Centre, Hospital S. Vito al Tagliamento, Pordenone, Italy

## Introduction

Migraine (M) is one of the most frequent diseases recognized as a cause of disability, with a relevant influence on the quality of life, particularly in women.

## Aim

Our study proposes to evaluate whether migraine affects reproductive choices and motherhood.

## Methods

We interviewed 399 women affected by M without (93.5%) and with (6.5%) aura, aged 17-79 years, using a semi-structured questionnaire. We collected data about intensity of attacks and frequency, fertility, pregnancies, abortion/miscarriage, number of children, influence on decisions about pregnancy and perceived ability in motherhood. A control group of 400 non migrainous women was interviewed for comparable items.

## Results

Among the 399 women interviewed, 94 (23.6%) were post-menopausal, while 305 (76.4%) had their menses; 155 (38.8%) were nulliparous while 244 (61.2%) had had at least one pregnancy; 224 (56.1%) were mothers while 175 (43.9%) did not have children; 86/244 (35.2%) experienced miscarriage or chose abortion at least once. Two hundred and twenty-three (55.9%) had less than 5 attacks/month, 130 (32.6%) had 5 to 14 attacks/month, 46 (11.5%) had 15 or more attacks/month. Pain intensity was low (NRS 1 to 4) in 18 (4.5%), moderate (NRS 5 to 7) in 108 (27.1%), high (NRS 8 to 10) in 273 (68.4%). Among the 399 women we interviewed, of those who wanted another child (184; 46.1%), 60/184 (32.6%) considered their headache a problem for a pregnancy. Two patients interrupted a sought-pregnancy because of the headache. Among patients who did not want (further) pregnancies (215; 53.9%), 20/215 (9.3%) listed headache among the causes for this choice. Few patients considered headache the only reason for their choice. Two hundred and twenty-seven of 399 women (56.9%) reputed headache an impediment to their being mothers. However, few women asked their doctor for information on the relationship between pregnancy and migraine.

## Conclusions

Our results seem to show a negative influence of M on decision of facing a pregnancy and self-perception as mothers, may be contributing in perceived disability.

Written informed consent to publication was obtained from the patient(s).

